# Identification and Characterization of Nine Novel X-Chromosomal Short Tandem Repeats on Xp21.1, Xq21.31, and Xq23 Regions

**DOI:** 10.3389/fgene.2021.784605

**Published:** 2021-11-17

**Authors:** Qinrui Yang, Jinglei Qian, Chengchen Shao, Yining Yao, Zhihan Zhou, Hongmei Xu, Qiqun Tang, Xiaoqin Qian, Jianhui Xie

**Affiliations:** ^1^ Department of Forensic Medicine, School of Basic Medical Sciences, Fudan University, Shanghai, China; ^2^ Department of Biochemistry and Molecular Biology, School of Basic Medical Sciences, Fudan University, Shanghai, China

**Keywords:** X-chromosomal STRs, multiplex PCR, recombination, linkage, kinship testing

## Abstract

The application of X-chromosomal short tandem repeats (X-STRs) has been recognized as a powerful tool in complex kinship testing. To support further development of X-STR analysis in forensic use, we identified nine novel X-STRs, which could be clustered into three linkage groups on Xp21.1, Xq21.31, and Xq23. A multiplex PCR system was built based on the electrophoresis. A total of 198 unrelated Shanghai Han samples along with 168 samples from 43 families was collected to investigate the genetic polymorphism and forensic parameters of the nine loci. Allele numbers ranged from 5 to 12, and amplicon sizes ranged from 146 to 477 bp. The multiplex showed high values for the combined power of discrimination (0.99997977 in males and 0.99999999 in females) and combined mean exclusion chances (0.99997918 and 0.99997821 in trios, 0.99984939 in duos, and 0.99984200 in deficiency cases). The linkage between all pairs of loci was estimated via Kosambi mapping function and linkage disequilibrium test, and further investigated through the family study. The data from 43 families strongly demonstrated an independent transmission between LGs and a tight linkage among loci within the same LG. All these results support that the newly described X-STRs and the multiplex system are highly promising for further forensic use.

## Introduction

On account of the distinct genetic characteristics and inheritance pattern, the weight of evidence provided by X-linked short tandem (STR) markers is enhanced in routine forensic practice. The application of X-chromosomal STRs (X-STRs) has been recognized a powerful tool in complex kinship testing, such as deficiency paternity cases and inbred cases, where the analysis of autosomal STRs may fail to give a clear conclusion (Szibor et al., 2003; Krawczak, 2007; Szibor, 2007; Pinto et al., 2011; Tillmar et al., 2017; Gomes et al., 2020). In some cases, the analysis of low-size X-STRs may provide greater statistical power than autosomal STRs in analyzing difficult samples such as skeletal human remains (Szibor, 2007). In addition, X-STR profiling could generate more informative evidence in individual identification using mixed stains containing female components compared with the detection results of autosomal STR loci.

To date, more than 50 X-STR loci have been identified and investigated for forensic practice (http://www.chrx-str.org). Several commercial kits have been developed for X-STR analysis (Tao et al., 2020; Xiao et al., 2021), and the most widely used are the Investigator Argus X-12 QS Kit (Hakim et al., 2021) and the AGCU X19 STR Kit (Zhang et al., 2016). Most commercial kits adopt X-STRs from four basic linkage groups (LGs) located on Xp22 (Hundertmark et al., 2008), Xq12 (Hering et al., 2006), Xq26, and Xq28 regions (Edelmann et al., 2008) according to clear linkage relationship. However, information provided by these X-STRs may not be efficient enough for specific cases, especially where mutational events or recombination events were observed within a linkage group (Hering et al., 2015).

Studies have been conducted to identify novel X-STR markers clustering other LGs on centromere (Edelmann et al., 2010), Xq21 (Szibor et al., 2005), and Xq22.1 (Edelmann et al., 2002), while attempts were made to add new LGs to the former four LGs to gain higher efficiency in detection. Nonetheless, it is noticeable that a relatively shorter physical distance could be observed between the linkage group on centromere and Xq12. A dispute over the linkage relationship between these two X-STR clusters—linked as one LG (Yang et al., 2019) versus independent as two LGs (Guo et al., 2019)—has been reported in previous studies. This would confuse the interpretation of X-STR profiling results in actual forensic practice.

In this study, in order to provide potential enhancement to the basic four LGs, we screened out nine novel X-STRs, which could be clustered into three novel LGs located >15 Mb from each other and from the basic four LGs. A multiplex PCR assay for STR typing was built based on the electrophoresis. A total of 198 unrelated Han Chinese individuals were collected to investigate the polymorphism and forensic parameters of the nine novel loci. The linkage between and within the three presumable LGs was estimated via Kosambi mapping function and linkage disequilibrium (LD) test, and further investigated through the family study.

## Materials and Methods

### Samples and DNA Extraction

Blood samples were collected from 198 unrelated Shanghai Han individuals (71 females and 127 males) and 43 families (*N* = 168). Informed consents were obtained from all participants prior to the sample collection. There are two types of families. Type One families were three-generation pedigrees comprising a grandfather, a mother, and a son. Type Two families were two-generation pedigrees comprising a father, a mother, and at least two offspring. When offspring are all males in a family, the sample of the father was not required for our study. The biological relationships of each family had been confirmed by paternity tests with autosomal STRs. Genomic DNA was extracted with the ReadyAmp Genomic DNA Purification System (Promega, United States). Typical control DNA of 2800M (Promega, United States) was used as reference in each single detection. This study was approved by the Ethics Committee of Fudan University.

### Short Tandem Repeat Search and Primer Design

The lobSTR (Gymrek et al., 2012) and data sets of Han Chinese in Beijing and Southern Han Chinese from the 1,000 Genomes Project were used to search for novel loci on the X chromosome (except for Xp22, Xq12, Xq26, and Xq28 regions). The criteria were as follows: 1) STR with 4- to 5-bp repeat motif, 2) more than five repeats, 3) more than three novel loci in the same region, 4) the power of discrimination in females higher than 0.5, and 5) easy-to-design primers. Genome build GRCh38 (hg38) was used to locate positions and flanking sequences. The primers for constructing a CE multiplex were designed using the PRIMER5 software.

### Genotyping and Sequence Analysis

DNA amplification of all samples was carried out with the Ex Taq DNA Polymerase (TaKaRa, China) according to the protocol of the manufacturer. All primers were pooled together with a final concentration of 1 µM. Thermal cycling followed the recommendation from the manufacturer: 30 cycles of denaturation at 98°C for 10 s, annealing at 55°C for 30 s, extension at 72°C for 60 s, and finally hold at 4°C. The PCR products were separated on an ABI 3130xl Genetic Analyzer (Applied Biosystems, United States) with the GeneScan 500 LIZ dye Size Standard (Applied Biosystems, United States). All alleles were determined with the GeneMapper ID v3.2 (Life Technologies, United States). To confirm the whole sequences of amplification products and locate polymorphic repeat regions, Sanger sequencing was performed using both forward and reverse reads.

### Statistical Analysis

The exact test for the Hardy–Weinberg equilibrium (HWE) using female data, and LD test using female and male data separately were estimated using the Arlequin v.3.5 software. Allele and haplotype frequencies, gene diversity (GD), haplotype diversity (HD), polymorphism information content (PIC), power of discrimination (PD), and the mean exclusion chances (MEC) in deficiency cases, trios, and duos (Krüger et al., 1968), MEC_Kishida (Kishida et al., 1997), MEC_Desmarais and MEC_Desmarais_Duos (Desmarais et al., 1998)] were calculated by the StatsX v2.0 software (Lang et al., 2019).

The genetic distances between STR pairs were estimated through a sequence-level genetic map constructed by Bjarni V. Halldorsson (Halldorsson et al., 2019). The Kosambi mapping function was applied to convert the cM genetic distance into a recombination fraction (Kosambi, 1943; Phillips et al., 2012). The maximum log odds (LOD) score analysis was carried out to investigate linkage between all pairs of loci (Yoo and Mendell, 2008). The recombination fraction was also calculated based on family data.

## Results

### Novel Short Tandem Repeat Information

**FIGURE 1:**
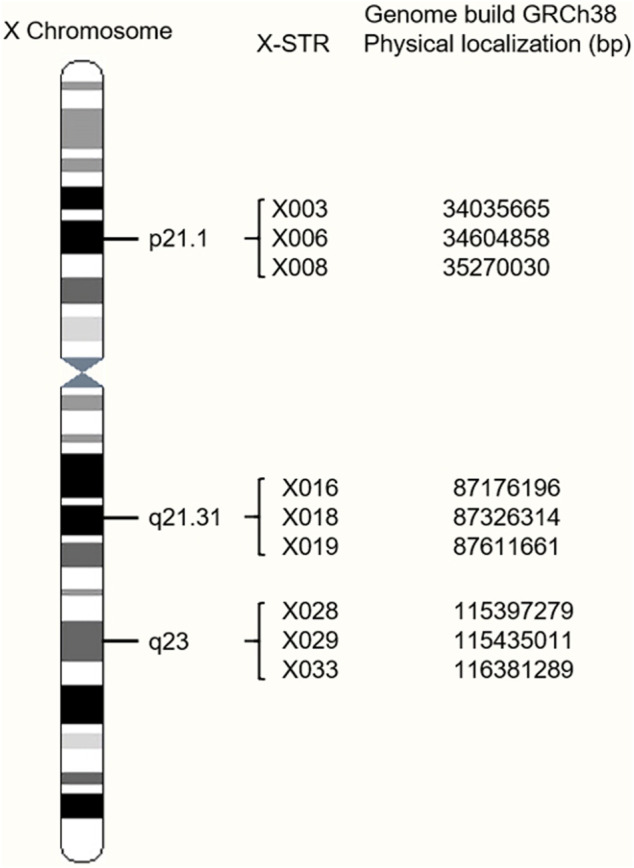
Location of the nine X-chromosomal short tandem repeats (X-STR) loci on an X-chromosome ideogram.

A total of nine novel X-STRs were screened out according to the criteria. An X-chromosome idiogram with details of the loci analyzed in this study is shown in Figure 1. The first marker group named LG1 in this study locates on Xp21.1 and spans approximately 0.763 cM. The second group named LG2 locates on Xq21.31 and spans approximately 0.417 cM. The third group named LG3 locates on Xq23 and spans approximately 1.906 cM. To assess the level of physical linkage statistically, we first computed the Kosambi recombination fraction within and between presumable LGs. The intragroup recombination fraction of LG1, LG2, and LG3 was 0.008, 0.004, and 0.019, respectively. The intergroup genetic distances were approximately 37.60 cM between LG1 and LG2, 25.67 cM between LG2 and LG3, and 62.85 cM between LG1 and LG3. The intergroup recombination fractions were 0.318 between LG1 and LG2, 0.236 between LG2 and LG3, and 0.425 between LG1 and LG3.

### Multiplex PCR

Primers for constructing a CE multiplex PCR are shown in Table 1. All the forward primers were labeled with fluorescent dyes: locus X003 and X016 were labeled with ROX; locus X006, X008, and X033 were labeled with FAM; locus X018 and X029 were labeled with HEX; locus X019 and X028 were labeled with TAMRA. Locus Amel was also included in the multiplex assay, and the forward primer was labeled with TAMRA. The amplification size ranged from 146 bp (X033) to 477 bp (X028) (Table 1). An example of the electropherogram of the positive control 2800M is shown in Supplementary Figure S1.

**TABLE 1:** Details of the multiplex system of the nine X-chromosomal short tandem repeats (X-STRs).

Linkage group	Locus	Primer sequence (5–3′)	Dye label	Product length	STR length min	STR length max	Allele range min	Allele range max
LG1	X003	F: ACA​TGC​AAC​TCT​TAT​GGA​TTA​CTA​T	ROX	214–238	44	68	11	17
		R: TGT​TGT​AAG​TGA​AAA​AGT​GGG​TTA						
LG1	X006	F: TGA​GCA​GAT​AAG​CAA​ATG​CAC​AGA	FAM	208–263	50	105	10	21
		R: CTG​GAA​GTG​GGA​TGG​AGT​GAA​AAC						
LG1	X008	F: CCC​TTT​GGA​TTT​CGT​TTT​TGA​G	FAM	275–291	28	44	7	11
		R: CTT​CTG​AAA​CCC​TGA​ATC​ATG​TA						
LG2	X016	F: ATT​CTG​TGT​AAC​TTC​AGA​GGG​AA	ROX	164–184	40	60	10	15
		R: CCT​AGA​AAT​GTG​CTT​TGA​CAG​GA						
LG2	X018	F: GCT​CCC​CTG​GTT​CTC​AAG​CTT​TTA	HEX	290–310	32	52	8	13
		R: CAA​GCT​AAA​GCT​GAA​AAG​CTG​ACT​T						
LG2	X019	F: TGA​CAT​TTT​GAT​CAA​TGA​AAA​ACC​GC	TAMRA	389–429	25	65	5	13
		R: GAG​CTT​TCA​TCT​CTG​CCT​TCT​TCT						
LG3	X028	F: CTG​CAG​TCC​AAG​CTA​CTC​TAC​TC	TAMRA	437–477	56	96	14	24
		R: AAG​GTT​TGC​ATC​ATA​TAA​TGG​CGT						
LG3	X029	F: GGA​AAG​CCA​GAG​TGA​CAT​CAT​TTT​A	HEX	372–400	24	52	6	13
		R: GTT​AGT​CTC​AAG​ATG​GAG​TCT​TTT​G						
LG3	X033	F: CTC​CAC​AGC​ACA​TTG​TTA​TTA​CTT	FAM	146–174	40	68	10	17
		R: GGC​AAC​AAC​ATG​GGT​GAA​AAG​A						

### Sequence Analysis

Using lobSTR, an estimate of the STR sequence structure was made by retrieving variations from datasets of Han Chinese in Beijing and Southern Han Chinese from the 1,000 Genomes Project. Loci X016, X019, and X029 were assumed to have simple repeat structures, while others were assumed to be complex STRs or compound STRs (Table 2). To confirm the whole sequences of amplification products and identify variable repeat regions for each locus, Sanger sequencing was performed. Although X008 and X018 were assumed as complex STRs by lobSTR, sequencing results showed that the variation was only observed in just one repeat region of the assumed repeat structure: the fifth repeat region with a motif of ATAG at X008 and the first repeat region with a motif of ATAG at X018. To simplify the nomenclature, they were defined as simple STRs. The allele nomenclature for X033 was also simplified. However, with sequence results, X033 was confirmed to have two different repeat structures: [TCTA]n and [TCTA]n [TCTG]1 [TCTA]n. According to the index of 1,000 genomes phase 3 (dbSNP 2.0 Build 154 v2), the second repeat structure was formed by a variation (rs782031781, A to G) in the last position of a motif. Modified repeat structures for the nine loci are shown in Table 2, as well as the STR sequences of the positive control 2800M.

**TABLE 2:** Sequence structure of the repeats of the nine novel X-STRs.

Locus	Assumed motif	Assumed STR sequence structure	Modified STR sequence structure	Genotype of 2800M	STR sequence of 2800M
X003	TCTA	[TCTA]n [TGTA]n [TCTA]n	[TCTA]n [TGTA]1 [TCTA]n	15	[TCTA]_11_ [TGTA]_1_ [TCTA]_3_
X006	GAAGA	[GAAGA]n [GAAAA]n [GAAGA]n	[GAAGA]_5_ [GAAAA]_1_ [GAAGA]n	20	[GAAGA]_5_ [GAAAA]_1_ [GAAGA]_14_
X008	ATAG	[ATAG]n [ATGG]n [ATAG]n [ATG]1 [ATAG]n [ATAA]n [ATAG]n	[ATAG]n	10	[ATAG]_10_
X016	ATAG	[ATAG]n	[ATAG]n	13	[ATAG]_13_
X018	ATAG	[ATAG]n [AGAT]n A [ATAG]n [ATAA]n [ATAG]n	[ATAG]n	11	[ATAG]_11_
X019	TACTA	[TACTA]n	[TACTA]n	7	[TACTA]_7_
X028	AAAG	[AAAG]n [AAGG]n	[AAAG]n [AAGG]n [AAAG]n	21	[AAAG]_18_ [AAGG]_2_ [AAAG]_1_
X029	AATA	[AATA]n	[AATA]n	10	[AATA]_10_
X033	TCTA	[TCTA]n T [TCTA]n [TTTG]n [TCTA]n [TCAT]n [CTGT]n [CTAT]n	[TCTA]n [TCTG]n [TCTA]n	12	[TCTA]_12_

Potential variations within flanking regions were also investigated, especially insertions and deletions (InDels), which would obstruct the allele nomenclature. Within the upstream flanking region of X018, an AC deletion (hg38: 87326311–87326312) was found in Shanghai Han samples. Alleles with the deletion were determined as N.2 by CE but modified to N + 1 based on the sequence results. In addition, the SNP rs621508 was found in the upper flanking region of X018. Allele A was observed in the reference sequence from GenBank while allele G was observed in all the sequenced Shanghai Han samples.

As the repeat structures were determined, the length of STR and the allele size were obtained (Table 1). The length of STR ranged from 24 bp (X029) to 105 bp (X006), and the allele ranged from 5 (X019) to 24 (X028).

### Hardy–Weinberg Equilibrium Test and Linkage Disequilibrium Test

Analysis of the HWE based on the female data is presented in Supplementary Table S1. The observed heterozygosity varied from 0.23944 (X016) to 0.91549 (X006) with an average of 0.68388, and expected heterozygosity varied from 0.51573 (X029) to 0.86914 (X006) with an average of 0.71293. Eight of the nine loci were found to be in HWE except X016 (*p* = 0.0000). It may be due to the small sample size. The pairwise exact test of LD was also performed. Significant associations were found in two pairs of loci after Bonferroni correction (*p* = 0.00138889): X016–X018 (0.00019802) and X018–X028 (0.00099009) (Supplementary Table S2, S3).

### Forensic Statistical Analysis

Allele frequencies and forensic parameters of the nine X-STRs are shown in Table 3. A total of 71 alleles were observed in the Shanghai Han population. The number of observed alleles ranged from 5 (X008) to 12 (X006). The lowest allele frequency of 0.0037 was observed at X016, X018, X028, and X029, while the highest allele frequency of 0.6097 was observed at X029. The GD and PIC ranged from 0.55270647 to 0.87163153 and 0.49208521 to 0.85562055, respectively. The PD spanned from 0.55065180 to 0.86839126 in males and from 0.73951960 to 0.96990843 in females. The MECs varied from 0.30538169 to 0.73972107 in deficiency cases (MEC_Krüger), 0.49208521 to 0.85562055 in trios (MEC_Kishida, MEC_Desmarais), and 0.34750737 to 0.75936208 in duos (MEC_Desmarais_Duos). All these showed that locus X006 was the most polymorphic marker in the Shanghai Han population.

**TABLE 3:** Allele frequencies and forensic parameters of the nine X-STRs and haplotypes.

Allele	X003	X006	X008	X016	X018	X019	X028	X029	X033	Combined
5						0.0186				
6						0.119		0.0074		
7			0.0186			0.4572		0.0149		
8			0.1599		0.0037	0.0372				
9			0.5465		0.1859	0.1152		0.6097		
10		0.026	0.2268	0.0223	0.4126	0.1784		0.2677	0.0781	
11	0.0112	0.0967	0.0483	0.2045	0.2825	0.052		0.0706	0.3346	
12	0.0297	0.1078		0.2193	0.1004	0.0149		0.026	0.2454	
13	0.4126	0.2454		0.4424	0.0149	0.0074		0.0037	0.1413	
14	0.3011	0.119		0.1078			0.0037		0.0372	
15	0.2268	0.0446		0.0037			0.0149		0.1041	
16	0.0112	0.0669					0.1152		0.0297	
17	0.0074	0.0409					0.2825		0.0297	
18		0.145					0.197			
19		0.0743					0.2007			
20		0.0297					0.1004			
21		0.0037					0.0409			
22							0.0149			
23							0.0186			
24							0.0112			
Allele count	7	12	5	6	6	9	11	7	8	
GD	0.68903553	0.87163153	0.62397225	0.70485797	0.70771122	0.72971167	0.81819275	0.55270647	0.79071099	
PIC	0.62802294	0.85562055	0.57101025	0.65607784	0.65474895	0.69756273	0.79076049	0.49208521	0.75944837	
PD_Male	0.68647406	0.86839126	0.62165265	0.70223768	0.70508032	0.72699899	0.81515114	0.55065180	0.78777155	
PD_Female	0.84325037	0.96990843	0.80621088	0.86517776	0.86269101	0.89603419	0.94144025	0.73951960	0.92663591	
MEC_Kruger	0.42292542	0.73972107	0.37614525	0.46116225	0.45643172	0.52479063	0.63774334	0.30538169	0.59645406	
MEC_Kishida	0.62802294	0.85562055	0.57113018	0.65607784	0.65474895	0.69744589	0.79076049	0.49208521	0.75956392	
MEC_Desmarais	0.62802294	0.85562055	0.57101025	0.65607784	0.65474895	0.69756273	0.79076049	0.49208521	0.75944837	
MEC_Desmarais_duo	0.48218233	0.75936208	0.42239785	0.51142369	0.51038631	0.55866141	0.671201009	0.34750737	0.63231176	
Haplotype count		74			52			72	
HD		0.98589299			0.96650846			0.98525603	
PIC		0.97770005			0.95747185			0.97702122	
PD_Male		0.97813006			0.95889816			0.97749811		0.99997977
PD_Female		0.99909169			0.99688433			0.99901677		0.99999999
MEC_Kruger		0.95649244			0.91831739			0.95554094		0.99984200
MEC_Kishida		0.97790425			0.95767949			0.97773645		0.99997918
MEC_Desmarais		0.97770005			0.95747185			0.97702122		0.99997821
MEC_Desmarais_duo		0.95710311			0.92068761			0.95573408		0.99984939

Note. PIC, polymorphism information content; PD, power of discrimination; MEC, mean exclusion chance; GD, gene diversity; HD, haplotype diversity.

### Haplotype Analysis

Haplotype frequencies were collected by StatsX v2.0 (Supplementary Table S1). A total of 74, 52, and 72 haplotypes were observed in LG1, LG2, and LG3. The most frequent haplotype (frequency) of each LG was 13-13-9 (7.87%) in LG1, 13-10-7 (11.81%) in LG2, and 17-9-12 (5.51%) in LG3. The biased haplotype frequencies suggested that loci were linked in respective LGs. Forensic parameters of LGs are presented in Table 3. The HD varied from 0.96650846 to 0.98589299. The PIC ranged from 0.95747185 to 0.97770005. The PD_Male and PD_Female spanned from 0.95889816 to 0.97813006, and 0.99688433 to 0.99909169. The MECs in duos spanned from 0.92068761 to 0.95710311, and the MECs in trios varied from 0.95747185 (MEC_Desmarais) to 0.97790425 (MEC_Kishida). The highest values were all observed in LG1, demonstrating the highest polymorphism among the three LGs. The combined power of discrimination in males and females in the Shanghai Han population were 0.99997977 and 0.99999999, respectively. The combined MECs were 0.99997918 and 0.99997820 in trios, 0.99984939 in duos, and 0.99984200 in deficiency cases (Table 3).

### Heredity of X-Chromosomal Short Tandem Repeats in Families

A total of 168 samples from 43 families (86 meiosis involved) were collected to investigate allele segregation of the nine X-STRs. Maternal haplotypes were identified successfully for each female sample. In this study, two inconsistencies were found to be one-step mutations (Table 4). In Family One, a paternal mutation from allele 16 to allele 17 was observed at the locus X006. The other one was a maternal mutation from allele 11 to allele 12 at the locus X018 in Family Two. Apparent intragroup recombination was observed in two families (Table 4). One occurred between the locus X003 and X006 (Family Three), where the mother was heterozygous at both loci, but two sons only inherited different alleles at the locus X003. In Family Four, although the intragroup recombination was evident within LG3, the location of the recombination breakpoint is unclear as the mother was homozygous at the locus X029. In addition, four more ambiguous X-STR transmissions (two at the locus X003, one at the locus X008, and one at the X018) were observed in this study (Table 4). They were explicable by either an intragroup recombination or a single-step mutation. The LOD score for each locus pair was also calculated and is shown in Table 5. LOD scores for all the intragroup locus pairs ranged from 5.14 to 9.79, and LOD scores between loci from different LGs were all less than 1. The recombination fraction for all pairs of loci was also calculated and is shown in Table 6. The recombination fraction for all pairs of intragroup loci varied from 0 to 0.065 and varied from 0.300 to 0.500 for all pairs of loci from different LGs.

**TABLE 4:** Heredity of X-STRs in families.

Mutation	Family One	X003			X006				X008		
	Father	15			16				10		
	Mother		15	17		12	18			9	9
	Daughter 1	15		17	**17**	12			10	9	
	Daughter 2	15	15		16		18		10		9
	Family Two	X016			X018				X019		
	Father	14			10				8		
		Mother		12	12		11	9			8	6
		Daughter	14	12		10	**12**			8	8	
		Son		12			11				8	
Intragroup recombination	Family Three	X003			X006				X008		
	Mother		13	15		11	17			9	9
	Son 1			15			17			9	
	Son 2		13				17			9	
	Family Four (A)	X028			X029				X033		
	Father	21			10				12		
	Mother		12	17		8	8			12	16
	Daughter 1	21	12		10		8		12		16
	Daughter 2	21	12		10	8			12	12	
	Family Four (B)	X028			X029				X033		
	Father	21			10				12		
	Mother		12	17		8	8			12	16
		Daughter 1	21	12		10	8			12		16
		Daughter 2	21	12		10	8			12	12	
	Ambiguous transmission	Family Five	X003			X006				X008		
		Mother		13	14		11	18			9	10
		Son 1		13			11				9	
Son 2				*13*			18				10
Family Six		X003			X006				X008		
Mother			13	14		12	17			9	10
Son 1			13				17				10
Son 2				*13*		12				9	
Family Seven		X003			X006				X008		
Mother			13	15		16	18			9	10
Son 1			13				18				10
Son 2			13				18				*9*
Family Eight		X016			X018				X019		
		Father	13			10				6		
		Mother		14	14		7	8			10	6
		Son		14			7					6
		Daughter	13	14		10	*8*			6		6

Note. One-step mutations are in bold and ambiguous transmissions are in italics.

**TABLE 5:** Log odds (LOD) scores for all pairs of loci.

Locus	X003	X006	X008	X016	X018	X019	X028	X029
X006	**6.08**							
X008	**5.61**	**6.84**						
X016	0.00	0.33	0.71					
X018	0.10	0.60	0.63	**5.93**				
X019	0.02	0.58	0.38	**9.79**	**6.55**			
X028	−0.50	0.42	−0.56	−0.51	−0.49	−0.35		
X029	0.16	−0.93	0.00	−0.40	−0.20	−0.27	**5.14**	
X033	−0.05	−0.03	−0.29	−0.24	−0.07	0.20	**5.70**	**5.14**

Note. Maximum LOD scores >3 are in bold.

**TABLE 6:** The recombination fraction for all pairs of loci.

Locus	X003	X006	X008	X016	X018	X019	X028	X029
X006	0.065							
X008	0.056	0.034						
X016	0.450	0.364	0.300					
X018	0.429	0.344	0.333	0.000				
X019	0.444	0.353	0.370	0.000	0.036			
X028	0.500	0.375	0.500	0.500	0.500	0.500		
X029	0.409	0.500	0.450	0.500	0.500	0.500	0.043	
X033	0.462	0.455	0.500	0.500	0.464	0.407	0.040	0.043

All the maternal haplotypes for the three LGs were informative as no haplotype was completely homozygous. Hence, recombination events between adjacent LGs could be identified directly. Intergroup recombination was observed in 29 families. The recombination fraction between LG1 and LG2, and between LG2 and LG3 were 0.366 and 0.415, respectively (Supplementary Table S4).

## Discussion

In this study, we identified nine novel X-STRs, which could be clustered into three LGs located on Xp21.1, Xq21.31, and Xq23. A multiplex PCR assay was built based on the electrophoresis. A total of 198 unrelated Shanghai Han samples along with 168 samples from 43 families were collected to investigate the polymorphism and forensic parameters. Allele numbers ranged from 5 to 12 and amplicon sizes ranged from 146 to 477 bp. The multiplex showed a great forensic efficiency as high values were achieved for the combined power of discrimination and combined MECs.

In kinship testing with X-STRs, the calculation of exact likelihood requires the consideration of linkage and LD (Krawczak, 2007). Thus, we utilized the Kosambi mapping function, the LD test, and a comprehensive family study to assess the tightness and strength of linkage between loci and between LGs. The intragroup recombination fraction of the Kosambi implied that the recombination may have the least chance to disrupt the haplotype of LG2, but a relatively higher chance in LG3. This was testified by the family data as the intragroup recombination was only observed in LG1 and LG3. However, unexpectedly, the intergroup recombination fractions of the Kosambi were quite different with the result generated from the actual family data. Since the recombination fractions obtained from the direct observation of STR transmissions in families is more trustworthy than that estimated from HapMap (Nothnagel et al., 2012), the paradox in our study may be reasonable and point to the existence of a potential hotspot between LG2 and LG3.

By LD test, the very limited LD and the suspicious association between loci X018 and X028 were observed. This may be due to the small sample size and the relatively low power of the LD tests (Tillmar et al., 2017).

In the family study, five ambiguous X-STR transmissions were uninformative for the investigation of the allele segregation. In Family Four, there were two plausible scenarios to illustrate how the recombination disrupted the linkage. In Scenario A, two daughters inherited different maternal alleles, and the intragroup recombination occurred between loci X028 and X029. Alternatively, in Scenario B, allele 8 of two daughters originated from the same maternal fragment, and the intragroup recombination occurred between loci X029 and X033. The other four ambiguous X-STR transmissions were explicable by either an intragroup recombination or a single-step mutation. For these cases, additional typing of flanking markers may provide further information (Nothnagel et al., 2012). LOD scores were calculated based on the family data. Significant linkage within each group was strongly supported by the fact that LOD scores for all the intragroup locus pairs were larger than 3. The pattern of free recombination between loci from different groups was also revealed as the LOD scores were less than 1. All these demonstrate that the newly described X-STRs and the multiplex system are highly promising for forensic use. Additionally, higher capability and efficiency of the system can be reached in the future as the remaining capacity in the multiplex system allows more loci to be included.

## Data Availability

The raw data supporting the conclusions of this article will be made available by the authors, without undue reservation.

## References

[B1] Desmarais D. Zhong Y. Chakraborty R. Perreault C. Busque L. (1998). Development of a Highly Polymorphic STR Marker for Identity Testing Purposes at the Human Androgen Receptor Gene (HUMARA). J. Forensic Sci. 43 (5), 1046–1049. 10.1520/jfs14355j 9729823

[B2] Edelmann J. Hering S. Kuhlisch E. Szibor R. (2002). Validation of the STR DXS7424 and the Linkage Situation on the X-Chromosome. Forensic Sci. Int. 125 (2-3), 217–222. 10.1016/s0379-0738(02)00005-1 11909667

[B3] Edelmann J. Hering S. Augustin C. Kalis S. Szibor R. (2010). Validation of Six Closely Linked STRs Located in the Chromosome X Centromere Region. Int. J. Leg. Med 124 (1), 83–87. 10.1007/s00414-009-0328-9 19229550

[B4] Edelmann J. Hering S. Augustin C. Szibor R. (2008). Characterisation of the STR Markers DXS10146, DXS10134 and DXS10147 Located within a 79.1kb Region at Xq28. Forensic Sci. Int. Genet. 2 (1), 41–46. 10.1016/j.fsigen.2007.08.001 19083788

[B5] Gomes I. Pinto N. Antão-Sousa S. Gomes V. Gusmão L. Amorim A. (2020). Twenty Years Later: A Comprehensive Review of the X Chromosome Use in Forensic Genetics. Front. Genet. 11, 926. 10.3389/fgene.2020.00926 33093840 PMC7527635

[B6] Guo J. Ji J. He G. Ren Z. Zhang H. Wang Q. (2019). Genetic Structure and Forensic Characterisation of 19 X-Chromosomal STR Loci in Guizhou Sui Population. Ann. Hum. Biol. 46 (3), 246–253. 10.1080/03014460.2019.1623911 31179782

[B7] Gymrek M. Golan D. Rosset S. Erlich Y. (2012). lobSTR: A Short Tandem Repeat Profiler for Personal Genomes. Genome Res. 22 (6), 1154–1162. 10.1101/gr.135780.111 22522390 PMC3371701

[B8] Hakim H. M. Khan H. O. Ismail S. A. Lalung J. Kofi A. E. Aziz M. Y. (2021). Population Data and Genetic Characteristics of 12 X-STR Loci Using the Investigator Argus X-12 Quality Sensor Kit for the Kedayan Population of Borneo in Malaysia. Int. J. Leg. Med 135 (4), 1433–1435. 10.1007/s00414-021-02577-0 33782746

[B9] Halldorsson B. V. Palsson G. Stefansson O. A. Jonsson H. Hardarson M. T. Eggertsson H. P. (2019). Erratum for the Research Article "Characterizing Mutagenic Effects of Recombination through a Sequence-Level Genetic Map. Science 363 (6425), 364. 10.1126/science.aaw8705 30679340

[B10] Hering S. Augustin C. Edelmann J. Heidel M. Dressler J. Rodig H. (2006). DXS10079, DXS10074 and DXS10075 Are STRs Located within a 280-kb Region of Xq12 and Provide Stable Haplotypes Useful for Complex Kinship Cases. Int. J. Leg. Med 120 (6), 337–345. 10.1007/s00414-005-0061-y 16344967

[B11] Hering S. Edelmann J. Haas S. Grasern N. (2015). Paternity Testing of Two Female Siblings with Investigator Argus X-12 Kit: A Case with Several Rare Mutation and Recombination Events. Forensic Sci. Int. Genet. Suppl. Ser. 5, E341–E343. 10.1016/j.fsigss.2015.09.135

[B12] Hundertmark T. Hering S. Edelmann J. Augustin C. Plate I. Szibor R. (2008). The STR Cluster DXS10148-Dxs8378-Dxs10135 Provides a Powerful Tool for X-Chromosomal Haplotyping at Xp22. Int. J. Leg. Med 122 (6), 489–492. 10.1007/s00414-008-0277-8 18688634

[B13] Kishida T. Wang W. Fukuda M. Tamaki Y. (1997). Duplex PCR of the Y-27H39 and HPRT Loci with Reference to Japanese Population Data on the HPRT Locus. Nihon Hoigaku Zasshi 51 (2), 67–69. 9184015

[B14] Kosambi D. D. (1943). The Estimation of Map Distances from Recombination Values. Ann. Eugenics 12, 172–175. 10.1111/j.1469-1809.1943.tb02321.x

[B15] Krawczak M. (2007). Kinship Testing with X-Chromosomal Markers: Mathematical and Statistical Issues. Forensic Sci. Int. Genet. 1 (2), 111–114. 10.1016/j.fsigen.2007.01.014 19083739

[B16] Krüger J. Fuhrmann W. Lichte K. H. Steffens C. (1968). On the Utilization of Erythrocyte Acid Phosphatase Polymorphism in Paternity Evaluation. Dtsch Z. Gesamte Gerichtl Med. 64 (2), 127–146. 4974841

[B17] Lang Y. Guo F. Niu Q. (2019). StatsX v2.0: the Interactive Graphical Software for Population Statistics on X-STR. Int. J. Leg. Med 133 (1), 39–44. 10.1007/s00414-018-1824-6 29564553

[B18] Nothnagel M. Szibor R. Vollrath O. Augustin C. Edelmann J. Geppert M. (2012). Collaborative Genetic Mapping of 12 Forensic Short Tandem Repeat (STR) Loci on the Human X Chromosome. Forensic Sci. Int. Genet. 6 (6), 778–784. 10.1016/j.fsigen.2012.02.015 22459949

[B19] Phillips C. Ballard D. Gill P. Court D. S. Carracedo Á. Lareu M. V. (2012). The Recombination Landscape Around Forensic STRs: Accurate Measurement of Genetic Distances between Syntenic STR Pairs Using HapMap High Density SNP Data. Forensic Sci. Int. Genet. 6 (3), 354–365. 10.1016/j.fsigen.2011.07.012 21871851

[B20] Pinto N. Gusmão L. Amorim A. (2011). X-chromosome Markers in Kinship Testing: A Generalisation of the IBD Approach Identifying Situations where Their Contribution Is Crucial. Forensic Sci. Int. Genet. 5 (1), 27–32. 10.1016/j.fsigen.2010.01.011 20457080

[B21] Szibor R. Hering S. Kuhlisch E. Plate I. Demberger S. Krawczak M. (2005). Haplotyping of STR Cluster DXS6801-Dxs6809-Dxs6789 on Xq21 Provides a Powerful Tool for Kinship Testing. Int. J. Leg. Med 119 (6), 363–369. 10.1007/s00414-005-0550-z 16096759

[B22] Szibor R. Krawczak M. Hering S. Edelmann J. Kuhlisch E. Krause D. (2003). Use of X-Linked Markers for Forensic Purposes. Int. J. Leg. Med 117 (2), 67–74. 10.1007/s00414-002-0352-5 12690502

[B23] Szibor R. (2007). X-chromosomal Markers: Past, Present and Future. Forensic Sci. Int. Genet. 1 (2), 93–99. 10.1016/j.fsigen.2007.03.003 19083736

[B24] Tao R. Zhang J. Xia R. Yang Z. Wang S. Zhang X. (2020). Genetic Investigation and Phylogenetic Analysis of Three Chinese Ethnic Groups Using 16 X Chromosome STR Loci. Ann. Hum. Biol. 47 (1), 59–64. 10.1080/03014460.2019.1704871 32064953

[B25] Tillmar A. O. Kling D. Butler J. M. Parson W. Prinz M. Schneider P. M. (2017). DNA Commission of the International Society for Forensic Genetics (ISFG): Guidelines on the Use of X-STRs in Kinship Analysis. Forensic Sci. Int. Genet. 29, 269–275. 10.1016/j.fsigen.2017.05.005 28544956

[B26] Xiao C. Yang X. Liu H. Liu C. Yu Z. Chen L. (2021). Validation and Forensic Application of a New 19 X-STR Loci Multiplex System. Leg. Med. 53, 101957. 10.1016/j.legalmed.2021.101957 34481193

[B27] Yang X. Chen Y. Zeng X. Chen L. Liu C. Liu H. (2019). Linkage, Recombination, and Mutation Rate Analyses of 19 X-Chromosomal STR Loci in Chinese Southern Han Pedigrees. Int. J. Leg. Med 133 (6), 1691–1698. 10.1007/s00414-019-02121-1 31317316

[B28] Yoo Y. J. Mendell N. R. (2008). The Power and Robustness of Maximum LOD Score Statistics. Ann. Hum. Genet 72 (Pt 4), 566–574. 10.1111/j.1469-1809.2008.00442.x 18410472 PMC2573393

[B29] Zhang Y.-D. Shen C.-M. Meng H.-T. Guo Y.-X. Dong Q. Yang G. (2016). Allele and Haplotype Diversity of New Multiplex of 19 ChrX-STR Loci in Han Population from Guanzhong Region (China). Electrophoresis 37 (12), 1669–1675. 10.1002/elps.201500425 27063464

