# Supplementary figures and images for: Identification and Characterization of Nine Novel X-Chromosomal Short Tandem Repeats on Xp21.1, Xq21.31, and Xq23 Regions
Source: Front Genet. 2021 Nov 17;12:784605. doi: 10.3389/fgene.2021.784605 (PMC8635773; doi:10.3389/fgene.2021.784605)

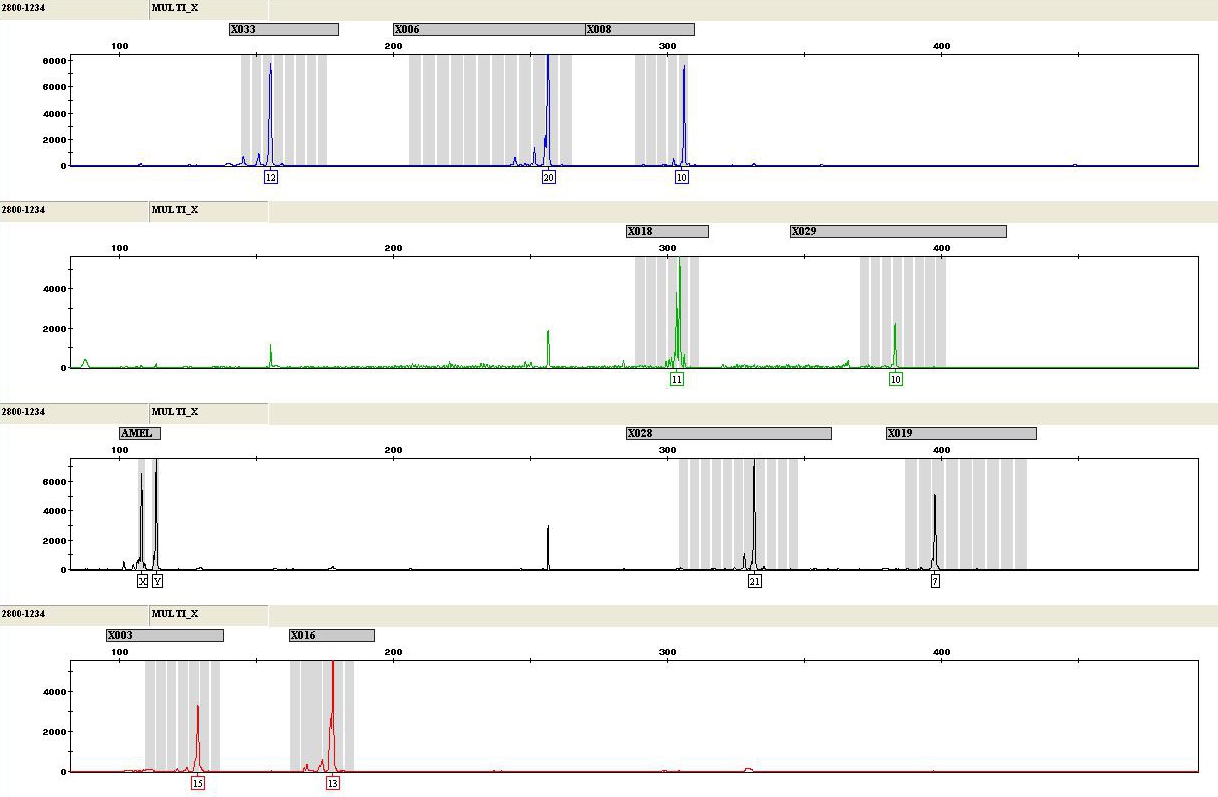

Supplement: Supplementary file 3 [file Image1.JPEG]
